# Delay and trace fear conditioning in a complex virtual learning environment—neural substrates of extinction

**DOI:** 10.3389/fnhum.2014.00323

**Published:** 2014-05-27

**Authors:** Heike Ewald, Evelyn Glotzbach-Schoon, Antje B. M. Gerdes, Marta Andreatta, Mathias Müller, Andreas Mühlberger, Paul Pauli

**Affiliations:** ^1^Department of Psychology, Biological Psychology, Clinical Psychology, and Psychotherapy, University of WürzburgWürzburg, Germany; ^2^Department of Clinical Psychology and Biological Psychology, School of Social Sciences, University of MannheimMannheim, Germany; ^3^Department of Experimental Psychology, Clinical Psychology and Psychotherapy, University of RegensburgRegensburg, Germany

**Keywords:** delay conditioning, trace conditioning, extinction, virtual reality, fMRI, prefrontal cortex

## Abstract

Extinction is an important mechanism to inhibit initially acquired fear responses. There is growing evidence that the ventromedial prefrontal cortex (vmPFC) inhibits the amygdala and therefore plays an important role in the extinction of delay fear conditioning. To our knowledge, there is no evidence on the role of the prefrontal cortex in the extinction of trace conditioning up to now. Thus, we compared brain structures involved in the extinction of human delay and trace fear conditioning in a between-subjects-design in an fMRI study. Participants were passively guided through a virtual environment during learning and extinction of conditioned fear. Two different lights served as conditioned stimuli (CS); as unconditioned stimulus (US) a mildly painful electric stimulus was delivered. In the delay conditioning group (DCG) the US was administered with offset of one light (CS+), whereas in the trace conditioning group (TCG) the US was presented 4 s after CS+ offset. Both groups showed insular and striatal activation during early extinction, but differed in their prefrontal activation. The vmPFC was mainly activated in the DCG, whereas the TCG showed activation of the dorsolateral prefrontal cortex (dlPFC) during extinction. These results point to different extinction processes in delay and trace conditioning. VmPFC activation during extinction of delay conditioning might reflect the inhibition of the fear response. In contrast, dlPFC activation during extinction of trace conditioning may reflect modulation of working memory processes which are involved in bridging the trace interval and hold information in short term memory.

## Introduction

Fear serves as an alert mechanism which is vital for survival, because adaptive fear reactions enable an individual to cope with survival threats by escaping or avoiding them. However, fear reactions can become maladaptive when they are no longer appropriate to the actual situation. The ability to readjust behavior is especially important in a rapidly changing environment. In anxiety disorders, this ability usually is impaired (e.g., Rauch et al., 2006; Schiller et al., 2012). The high prevalence of anxiety disorders (~14% within 12 months; BGS98 2000) has led to extensive research in the field of fear and the neural systems involved in fear learning and its extinction.

Fear learning in animal and human research is mainly examined on the basis of Pavlovian fear conditioning (Pavlov, 1927). In this paradigm, an initially neutral conditioned stimulus (CS), such as a tone or a light, is paired with an aversive unconditioned stimulus (US), such as a shock. After several pairings, the CS is associated with the US and evokes a conditioned fear response (CR) on its own. In differential fear conditioning, there are two initially neutral stimuli. One of them, the CS+, is paired with the aversive US, while the other one, the CS−, is not. In this case, only the CS+ elicits a CR after several pairings with the US. Based on timing aspects of the CS–US pairing we have to distinguish two forms of classical fear conditioning: In delay conditioning, the US follows the CS with no temporal gap (e.g, either the US directly follows the CS or the CS and the US coterminate meaning that there is a overlap in time), while in trace conditioning there is a temporal gap, called the trace interval, between CS offset and US onset. In the latter case, a “memory trace” is necessary to learn the association between the CS and the US (Pavlov, 1927).

This small difference in timing was found to be associated with differences in the neural structures underlying the acquisition of fear. The most prominent structure reported to be crucial for fear learning is the amygdala. Information about the CS and the US is transmitted from sensory cortices via the thalamus to the amygdala, which controls the expression of the fear reaction via projections to the brainstem (e.g., LeDoux, 2000). Other areas found to be involved in fear acquisition in delay conditioning are the anterior cingulate cortex (ACC) and the insular cortex. Activation in the ACC has been linked to the anticipation of pain. For example, Büchel et al. (1998) assumed that the ACC, together with activation of the anterior insula, might integrate nociceptive input with memory and therefore allows for appropriate responses to subsequent stimuli, as has been shown in pain studies (e.g., Coghill et al., 1994). The insula has been reported to be involved in conveying a cortical representation of fear to the amygdala (Phelps et al., 2001). Knight et al. (2004) and Milad et al. (2007) proposed that the ACC is involved in the expression of the fear response. It has often been reported that the hippocampus is involved in fear conditioning. The current opinion is, that contextual information, which can be of spatial or temporal nature, is represented in the hippocampus (O'Keefe and Dostrovsky, 1971). Hippocampal activation has been shown to be crucial for trace conditioning since the trace interval requires the formation of a temporal context. This has been found both in fear conditioning (Büchel et al., 1999; Knight et al., 2004) as well as in eyeblink conditioning (Cheng et al., 2008). Also, Clark et al. (2002) reported that trace eyeblink conditioning additionally depends on the hippocampus and the neocortex, whereas for delay eyeblink conditioning activations of the cerebellum and the brainstem are sufficient. Human delay fear conditioning has been reported to occur without explicit hippocampal activity, too (see e.g., LaBar et al., 1998; Phelps et al., 2004; Schiller et al., 2008). Taken together, we conclude that the main neural structures involved in delay fear conditioning are the amygdala, the insula, and the ACC (see review by Sehlmeyer et al., 2009). However, for both fear and eyeblink trace conditioning, declarative memory, which is associated with hippocampus activity, is formed additionally.

To understand the mechanisms of fear and the development and maintenance of anxiety disorders, it is not sufficient to study the acquisition of fear only. Inappropriate fear can also result from a deficit in extinction of the fear (Baas et al., 2008). Extinction occurs if the CS is presented several times without the US. Importantly, there is convincing evidence that extinction does not lead to forgetting or unlearning, but rather to the formation of a new memory inhibiting the acquired fear memory (Bouton, 2002, 2004; Milad and Quirk, 2002; Myers and Davis, 2002; Quirk, 2002). To date, the neural structures involved in the extinction of fear are less understood than those involved in fear acquisition. The amygdala has been shown to play an important role in both acquisition and extinction. In addition, it is assumed that, as extinction, i.e., new learning, takes place, the prefrontal cortex inhibits the expression of conditioned fear (Quirk et al., 2006). Rodent models of extinction suggest that during extinction, an inhibitory memory trace between ventromedial prefrontal cortex (vmPFC) and amygdala is established (Sotres-Bayon et al., 2004, 2007), by means of which the expression of fear is inhibited. The vmPFC activates GABAergic intercalated cells in the amygdala which in turn inhibit the central nucleus of the amygdala (Quirk et al., 2006; Sotres-Bayon et al., 2007). Evidence for this model has been provided by lesion studies (Morgan and LeDoux, 1993; Quirk et al., 2000; Morgan et al., 2003; Lebron et al., 2004). For example Morgan and LeDoux (1993) showed that rats with lesions of the medial PFC were resistant to extinction learning in a delay fear conditioning paradigm. Human studies on the extinction of fear memory acquired through classical delay conditioning have confirmed the role of the amygdala and the vmPFC (Phelps et al., 2004; Milad et al., 2007). The insula and the ACC have also been shown to be involved in extinction learning in humans (Gottfried and Dolan, 2004; Phelps et al., 2004). Additionally, extinction learning was found to be highly context dependent. Kalisch et al. (2006) for example showed that a network containing the vmPFC and the hippocampus is activated during context dependent recall of extinction memory.

The striatum seems to be involved in affective learning, too, more precisely in the processing of prediction errors which occur when the expected result does not match the actual result (e.g., Delgado et al., 2008). Besides clear evidence that the striatum is involved in appetitive conditioning (e.g., O'Doherty et al., 2001), there is also growing evidence for its involvement in the processing of prediction errors in aversive conditioning such as classical fear conditioning (Jensen et al., 2003; Delgado et al., 2008). Importantly, the absence of the US during extinction resembles a positive prediction error, meaning that a negative outcome, which is expected, does not occur. Thus, the striatum has to be considered as an important region in the extinction of conditioned fear.

Imaging studies examining extinction so far focused on delay conditioning, and little is known about extinction following trace conditioning in humans. Since differences in involved brain regions have been found during acquisition of delay and trace fear conditioning (see discussion above) it is likely that different brain areas are involved during extinction, too. Many situations in real life, in which fear is acquired, are closer to the trace than the delay conditioning paradigm, since there is often a temporal gap between a CS and the predicted US. Given the relevance of fear extinction for the maintenance of anxiety disorders, it seems very important to detect possible differences in the neural networks involved in these different types of fear conditioning.

Based on prior results in humans, we expected that extinction of delay and trace fear conditioning is associated with activations of the amygdala and the vmPFC as well as involvement of the insular and the anterior cingulate cortices and the striatum. In contrast, we assumed differences between delay and trace conditioning regarding activations in the prefrontal cortex. One model of the functional organization of the lateral PFC postulates that the ventrolateral part of the PFC is mainly involved in the maintenance of information, such as retaining a sequence of letters in working memory, whereas the dorsal part is more important when it comes to manipulation of information, such as reordering the sequence into alphabetical order (D'Esposito et al., 1999). There are also findings that nonhuman primates and humans with lesions of the dorsolateral PFC (dlPFC) are less able to adjust behavior appropriately in delayed response tasks (e.g., D'Esposito et al., 2000). In these tasks it is necessary to hold information in working memory over a short temporal gap before making choices and decisions. This demand is similar to forming and changing a memory trace bridging the trace interval in trace fear conditioning. Therefore, in contrast to vmPFC contributions to the extinction of delay memory, the dlPFC might—exclusively or additionally—be involved in the extinction of trace memory. Evidence for the involvement of the dlPFC in trace eyeblink conditioning also comes from animal models. Weiss and Disterhoft (2011) propose a neural network in which the dlPFC, together with prelimbic areas, orchestrates neural activity that bridges the trace interval. Finally, since the hippocampus is assumed to be involved in the formation of declarative memory in a conditioning process, we expected it to play a greater role in the extinction of trace memory compared to delay memory.

The present study used virtual reality (VR) to implement both the delay and the trace fear conditioning paradigm. VR is a powerful tool for studying fear reactions in ecologically valid environments (Mühlberger et al., 2007a,b, 2008a,b). It has successfully been applied in treatment of specific phobias (Mühlberger et al., 2006) as well as in conditioning studies (Baas et al., 2004, 2008; Alvarez et al., 2008; Glotzbach et al., 2012; Tröger et al., 2012). The use of VR allows for the full control of a fear conditioning situation that is closer to the complexity of learning situations in real life than most laboratory designs. The used virtual environment consisted of a corridor and an office, through which subjects were passively guided while lying in the scanner. In both the delay (DCG) and the trace conditioning group (TCG), a blue and a yellow light in the office served as CS+ and CS−, respectively, and a mildly painful electric stimulus as US. Besides ratings of valence, arousal, fear, and contingency assessed after acquisition and extinction phases, the BOLD response differences between the CS+ and CS− served as indices of brain responses related to learning. Specifically, differences between delay and trace fear conditioning during extinction were analyzed. Bold responses during acquisition were not analyzed because of an overlap of brain responses to the CS and US in the learning phase due to their temporal closeness.

## Methods and materials

### Participants

The final sample consisted of 26 participants, 13 in the DCG (5 male, 8 female, mean age = 23.1 years, *SD* = 3.0 years) and 13 in the TCG (4 male, 9 female, mean age = 23.5 years, *SD* = 2.5 years). All participants gave their written informed consent and received 12 €/h for participation. The study protocol was approved by the Ethics Committee of the Medical Faculty of the University of Würzburg.

To reach this sample, a total of 43 right-handed volunteers (14 male, 29 female; age 19–29) had to be recruited. They were randomly assigned to the DCG or the TCG. Excluding criteria were past or present psychiatric disorders, use of antipsychotic drugs, regular alcohol or drug consumption (one subject was excluded), and allochromasia (for blue and yellow) assessed by self-report. Twelve subjects had to be excluded due to technical problems, and one subject because of extensive head movements during scanning. Three participants did not explicitly learn the contingency between the CSs and the US (only DCG, see below). They were also excluded because the small group size did not allow for a separate analysis to investigate distinct neuronal patterns in aware vs. unaware participants.

### Stimuli and apparatus

#### VR environment

**Figure 1:**
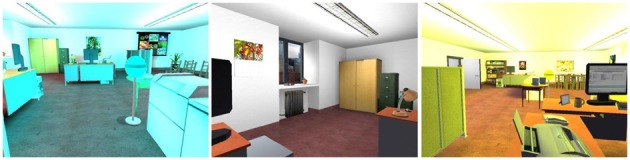
**Virtual office illuminated in two different lights (right in blue and left in yellow) which served as conditioned stimuli (CS) and with normal illumination (ISI, inter-stimulus interval)**.

For creating the virtual environment of the experiment we used the Source Engine SDK (Valve Corporation, Bellevue, Washington, USA). Some office models were used from the free Source Engine Modification “Weekday Warrior” (http://www.moddb.com/mods/weekday-warrior). The virtual environment consisted of an office and an associated corridor evenly illuminated in a neutral white light. In the office, a lamp situated in the middle of the room could be switched on and off. The color of the light illuminated by the lamp was either blue or yellow and served as CS+ or CS−, respectively (see Figure 1). If turned on, the lamp illuminated the whole room. One light (CS+) was followed by a mildly painful electric stimulus (US) with 100% contingency; the other light (CS−) was never followed by an US. Colors of CS+ and CS− were counterbalanced across participants and groups. In the delay conditioning paradigm, the lights were always switched on for 8 s, and the US was presented simultaneously with the offset of the CS+. In the trace paradigm, the lights were presented for 4 s, and the US was presented 8 s after CS+ onset; thus, the trace interval between CS and US lasted 4 s. To avoid movement in the scanner and enhance control over the course of events during the experiment, participants were guided through the VR environment on a prerecorded path. We used the in-house written VR simulation software CyberSession to manipulate the VR environment during the experiment (e.g., switching on the light, delivering the electrical pulse). VR rendering was done by an image generator running the in-house written Source SDK modification VRSessionMod 0.3. The virtual environment was displayed via MRI-compatible goggles (VisuaStim; Magnetic Resonance Technologies, Northridge, CA, USA).

#### Electric stimuli

The US was a mildly painful electric stimulus generated by a current stimulator (Digitimer DS7A, Digitimer Ltd, Hertfordshire, England). It was delivered at the left index finger through surface bar electrodes consisting of two durable gold-pasted stainless steel disk electrodes with 9mm diameter, 30mm spacing, and with an impedance of max. 5 Ω. Electric stimuli associated with the CS+ were triggered automatically by CyberSession during conditioning for 200 ms with a frequency of 50 Hz. Current intensity was determined individually for each participant in the beginning of the experiment (for a detailed description of the adjustment of individual current intensity see Andreatta et al., 2010). It was adjusted at the individual pain threshold and increased by 30% to prevent habituation to the US. Both conditioning groups did not differ in current intensity (delay group: *M* = 2.25 mA, *SD* = 0.99; trace group: *M* = 2.18, *SD* = 0.90), *t*_(23)_ = 0.19, *p* = 0.853, and pain ratings (delay group: *M* = 5.00, *SD* = 0.84; trace group: *M* = 5.04, *SD* = 1.57), *t*_(23)_ = −0.08, *p* = 0.934, of the US.

### Psychometric measures

#### Ratings

At several times during the experiment, ratings of valence (from “very negative” to “very positive”), arousal (from “not arousing at all” to “very arousing”), fear (from “no fear” to “extreme fear”), and CS–US contingency (from “not likely at all” to “very likely”) were collected, each on scales from 0 to 100.

#### Awareness

Explicit knowledge of contingencies between CS and US was assessed on the basis of the question “During which light presentation did you receive electric shocks?” Participants who were able to state the correct color of light after the second acquisition run were labeled “aware,” the others were labeled “unaware.” While 26 participants met these criteria of awareness; three participants in the DCG failed and were labeled “unaware.” There were no unaware participants in the TCG.

### Procedure

After reading information about the scanning procedure, participants completed the questionnaires on personal information and excluding criteria. Then they received written instructions related to the experiment and gave their written informed consent. The electrode for electric stimulation was attached when participants were already positioned in the scanner room. After that, the individual pain threshold was determined and adjusted as explained earlier. This preparation phase was followed by a pre-acquisition block, in which participants were guided through the virtual office once to get used to the environment and the two different lights (each light was presented once). After that, participants were told that they would be able to predict the electric stimuli if they paid close attention to the experiment.

The following acquisition phase consisted of two blocks. Each block included two passages through the office. During one passage, participants were guided through the office once and were exposed to four CS+ and four CS−. The CS+ was always followed by the US. One room visit lasted 172 s; accordingly one block lasted approximately 6 min. During the complete acquisition participants were exposed to 16 CS+ and 16 CS− and received 16 US.

The extinction phase consisted of one block including two visits with the same duration and CS frequencies as the acquisition trials (i.e., 8 CS+ and 8 CS−). No US was applied during extinction.

Classification of lights as CS+ and CS− as well as order of stimuli was pseudo-randomized across participants. In total, there were four different courses of events, two of them with the blue light and two with the yellow light serving as CS+. The length of the interstimulus interval (ISI) was also pseudo-randomized and varied between 11 and 13 s in steps of 250ms.

### Ratings

After each of the two acquisition blocks, awareness was measured by posing free recall questions as described above. Participants rated screenshots of the room with either the CS+ or the CS− light switched on regarding valence, arousal and fear after pre-acquisition, each acquisition block and extinction. Additionally, after both acquisition phases and extinction, contingency of CS+ and CS− with the US was measured. For all ratings, questions and screenshots were presented via the goggles. Participants were told to relate their answers to the way they felt during the last phase of the experiment. Answers were given orally via the speaker system of the scanner room and recorded by the investigator.

### Magnetic resonance imaging

A 1.5-T whole-body magnetic resonance tomograph (MagnetomAvanto, SiemensHealthcare, Erlangen, Germany) with standard 12-channel head coil and integrated head holder was used for acquisition of structural and functional brain images. Structural imaging consisted of 160 T1-weighted sagittal magnetization-prepared rapid gradient-echo imaging (MP-RAGE) 3D MRI sequence (MPRAGE, 1mm slice thickness, TR = 2250 ms, TE = 3.93 ms, flip angle: 8°, FOV: 256mm, matrix: 256 × 256, voxel size: 1 × 1 × 1mm^3^). The acquisition of structural images was situated at the end of the experiment.

Functional imaging was conducted in four phases (pre-acquisition, first and second acquisition phase and extinction). During subjective ratings after each of the experimental phases, imaging was intermitted. A total of 161 volumes was registered using a T^*^2-weighted gradient echo-planar imaging sequence (EPI) with 25 axial slices [slice thickness 5-mm with 1-mm gap, interleaved (descending) order] covering the whole brain (TR: 2500 ms; TE: 40 ms; flip angle: 90°; FOV: 240 × 240 mm; matrix size: 64 × 64; voxel size: 3.1 × 3.1 × 3mm^3^) for functional imaging. Orientation of axial slices was parallel to the AC-PC line. The first 8 images of each phase were excluded from analysis to allow for T1 equilibration.

### Image preprocessing and statistical analysis

#### Imaging

Analysis of fMRI data was performed with Statistical Parametric Mapping (SPM8, Wellcome Department of Cognitive Neurology, London) integraded in MatLab 7.0 (Mathworks Inc., Sherborn, MA). After slice time correction, functional images were realigned. T1-scans were coregistered to each participant's mean image of the realigned. The mean functional images were normalized to the Montreal Neurological Institute (MNI) single-subject template (Evans et al., 1992). Normalization parameters obtained from the previous segmentation procedure of coregistered T1 images were applied and images were resampled (voxel size 2 × 2 × 2mm^3^). Subsequently, EPI images were spatially smoothed with an 8-mm full-width-half-maximum (FWHM) Gaussian kernel and filtered with a 128 ms high pass filter.

The different experimental conditions were modeled using a boxcar reference vector convolved with a canonical hemodynamic response function (general linear model, Kiebel and Holmes, 2003). The six movement parameters of the rigid body transformation, applied by the realignment procedure, were included to regard variance caused by residual movement. Low-frequency signal drift was filtered using a first-order autoregressive model. Parameter estimates were subsequently calculated for each voxel using weighted least squares to provide maximum likelihood estimates based on the non-sphericity assumption in order to get identical and independently distributed error terms. Since we were especially interested in the activation related to the extinction of fear reactions, the extinction phase served as main test phase and the BOLD signal was calculated at the onset of the colored lights. In a conditioning paradigm with a CS–US contingency of 100% during acquisition we expected rapid decrease of fear reactions. To account for this, the extinction phase was divided into two parts of equal duration and the first and second half were analyzed separately (early and late extinction). In a second step we compared activation during early extinction (first to fourth CS+) with activation during late extinction (fifth to eighth CS+).

First level individual contrast images (CS+ > CS−) were used in a second-level analysis (one sample *t*-test). Conditioning groups (delay and trace) were analyzed separately for the contrast CS+ > CS−. We also analyzed the contrast early extinction (CS+ > CS−) > late extinction (CS+ > CS−). ROI analyses were carried out for the amygdala, the hippocampus, the insula, the ACC (Brodmann areas 24, 32, and 33) the striatum (caudate and putamen) and the ventromedial (medial orbital frontal gyrus) and dorsolateral prefrontal cortices (middle frontal gyrus) at an uncorrected threshold of *p* = 0.005 with a minimum cluster size of ten voxel. Additionally, we conducted an explorative whole brain analysis which also included the cerebellum (*p* = 0.001, uncorrected, minimum cluster size of five voxel). ROIs were based on masks of the WFU Pick Atlas (Maldjian et al., 2003) and Brodmann Areas (BA).

#### Ratings

For the ratings of valence, arousal, and fear, ANOVAs were conducted for pre-acquisition and extinction with the between factors stimulus (CS+, CS−) and group (delay, trace). Ratings during acquisition were analyzed with repeated measures ANOVAs with the between factors stimulus (CS+, CS−) and group (delay, trace) and the additional within factor phase (Acquisition 1, Acquisition 2). Contingency ratings were not collected after pre-acquisition and thus were only analyzed for acquisition and extinction.

All rating data were analyzed using SPSS for Windows (Release 17.0). Alpha was set at.05 for all statistical tests, effect sizes are reported as η^2^_*p*_ scores.

## Results

### Ratings

#### Pre-acquisition

As expected, after the pre-acquisition phase, CS+ and CS− did not differ in valence, arousal, or fear in any group (all *p*s > 0.23).

#### Acquisition

***Valence ratings***. For valence ratings, we found a significant main effect of stimulus, *F*_(1, 24)_ = 13.49, *p* = 0.001, η^2^_*p*_ = 0.360, as well as a marginally significant interaction of Phase × Stimulus, *F*_(1, 24)_ = 3.92, *p* = 0.059, η^2^_*p*_ = 0.140. The CS+ was rated overall more negative than the CS− (CS+: *M* = 36.83, *SD* = 20.49; CS−: *M* = 65.38, *SD* = 27.88). Significant effects involving the factor group were not detected.

***Arousal rating***. The analysis revealed a significant main effect of stimulus, *F*_(1, 24)_ = 33.05, *p* < 0.001, η^2^_*p*_ = 0.579, and a significant three way interaction of Phase × Stimulus × Group, *F*_(1, 24)_ = 4.30, *p* = 0.049, η^2^_*p*_ = 0.152. After the first acquisition phase, the CS+ elicited more arousal than the CS− in both the delay group, *t*_(12)_ = 2.578, *p* = 0.024 (CS+: *M* = 40.38, *SD* = 25.70; CS−: *M* = 14.62, *SD* = 19.84), and the trace group, *t*_(12)_ = 5.41, *p* < 0.001 (CS+: *M* = 51.15, *SD* = 28.88; CS−: *M* = 8.46, *SD* = 9.87). After the second phase, we found a similar pattern as after the first phase in the trace group, *t*_(12)_ = 4.38, *p* = 0.001 (CS+: *M* = 40.77, *SD* = 26.91; CS−: *M* = 6.92, *SD* = 9.47), while in the delay group the effect was more pronounced than after the first phase, *t*_(12)_ = 3.534, *p* = 0.004, (CS+: *M* = 43.46, *SD* = 27.03; CS−: *M* = 9.62, *SD* = 18.76).

***Fear ratings***. For fear ratings, we again found a significant main effect of stimulus, *F*_(1, 24)_ = 22.32, *p* < 0.000, η^2^_*p*_ = 0.482, indicating that the CS+ elicited overall more fear than the CS− in both groups (CS+: *M* = 36.25, *SD* = 30.40; CS−: *M* = 5.58, *SD* = 11.57).

***Contingency ratings***. The CS+ was clearly perceived as more likely to be followed by an electric stimulus during acquisition, main effect of stimulus, *F*_(1, 24)_ = 201.45, *p* < 0.001, η^2^_*p*_ = 0.894 (CS+: *M* = 88.65, *SD* = 18.72; CS−: *M* = 9.62, *SD* = 14.69). Additionally, we found a significant interaction of Phase × Stimulus, *F*_(1, 24)_ = 5.66, *p* = 0.026, η^2^_*p*_ = 0.191. Already after the first phase, the CS+ was rated as more likely to be followed by the US than the CS−, *t*_(25)_ = 6.681, *p* < 0.001 (CS+: *M* = 81.73, *SD* = 29.29; CS−: *M* = 15.00, *SD* = 27.75), but after the second phase this difference between CS+ and CS− further increased, *t*_(25)_ = 26.239, *p* < 0.001 (CS+: *M* = 95.58, *SD* = 14.45; CS−: *M* = 4.23, *SD* = 11.38). The contingency between the CS+ and the US was rated higher after the second than after the first phase, *t*_(25)_ = −2.612, *p* = 0.015.

#### Extinction

***Valence ratings***. The CS+ and the CS− did no differ significantly in their valence after extinction, *F*_(1, 24)_ = 3.48, *p* = 0.075, η^2^_*p*_ = 0.127, although a marginal difference was still present.

***Arousal ratings***. The analysis revealed a significant main effect of stimulus, *F*_(1, 24)_ = 20.30, *p* < 0.001, η^2^_*p*_ = 0.458, as well as a significant interaction of Stimulus × Group, *F*_(1, 24)_ = 5.26, *p* = 0.050, η^2^_*p*_ = 0.151. In the delay group, arousal ratings of CS+ and the CS− did not differ significantly. However, in the trace group, the CS+ was still rated as more arousing than the CS−, *t*_(12)_ = 4.368, *p* = 0.001 (CS+: *M* = 21.76, *SD* = 6.03; CS−: *M* = 10.05, *SD* = 2.91).

***Fear ratings***. The main effect of stimulus was still significant after the extinction phase, *F*_(1, 24)_ = 11.61, *p* = 0.002, η^2^_*p*_ = 0.326. We also found a marginal interaction of Stimulus x Group, *F*_(1, 24)_ = 3.76, *p* = 0.064, η^2^_*p*_ = 0.136, suggesting similar results as for arousal ratings: In the trace group, the CS+ was after extinction still associated with more fear than the CS−, *t*_(12)_ = 3.726, *p* = 0.003 (CS+: *M* = 25.77, *SD* = 21.39; CS−: *M* = 6.15, *SD* = 10.44), while there was no such difference in the delay group, *t*_(12)_ = 1.054, *p* = 0.313.

***Contingency ratings***. As for fear ratings, the main effect of stimulus persisted during the extinction phase, *F*_(1, 24)_ = 18.39, *p* < 0.001, η^2^_*p*_ = 0.434. After extinction, the CS+ was still more associated with the US than the CS− (CS+: *M* = 47.31, *SD* = 35.98; CS−: *M* = 12.31, *SD* = 20.06).

In sum, arousal and fear ratings suggest that extinction proceeded more slowly in the trace group compared to the delay group. After extinction, the trace group rated the CS+ still as more arousing and more frightening than the CS−, whereas in the delay group these differences were no longer present after extinction.

### Imaging data

#### Early extinction

**Figure 2:**
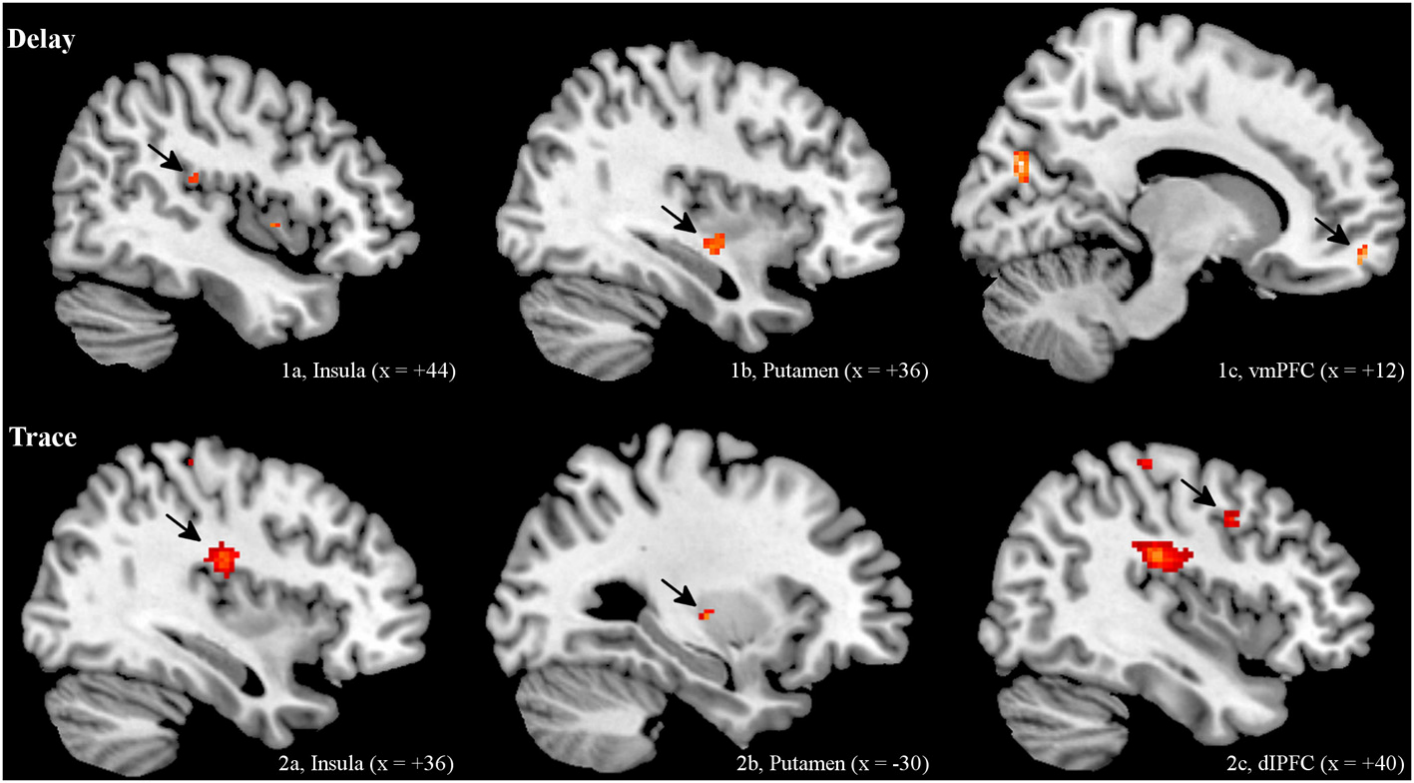
**BOLD Signals (CS+ > CS−) during early extinction (ROI, α < 0.005, uncorrected)**. In both DCG and TCG, Insula and Putamen were activated during early extinction. In the DCG, we observed significant activation of the vmPFC (medial orbital frontal gyrus R), while in the TCG the dlPFC (middle frontal gyrus R) was significantly activated.

***ROI analysis***. Regarding the contrast of CS+ minus CS−, both groups showed insular and striatal activation during early extinction. Interestingly, the two groups differed in their prefrontal activation (see Figure 2). In the DCG, we observed significant activation of the vmPFC (medial orbital frontal gyrus R), while in the TCG the dlPFC was significantly activated (middle frontal gyrus R). Additionally, the trace group showed activation of the dorsal part of the ACC (BA 33).

***Whole brain analysis***. In addition to the areas defined as ROIs, we also found significant activation in several other regions. In the DCG, the cuneus (L), the left motor cortex (precentral gyrus L), and the middle occipital gyrus (R) were activated. In the TCG, we found activations in the somatosensory cortex (postcentral gyrus L), the calcarine (R), the rolandic operculum (R), and the ventral ACC (middle cingulate cortex L, BA 24).

For exact coordinates see Table 1.

**Table 1:** **Significant activations revealed by whole brain (WB) and regions of interest (ROI) analysis for contrast CS+ > CS− during early extinction**.

**Group**	**Brain structure**	***x***	***y***	***z***	***Z***	**Cluster size**	***P***
Delay	Cuneus R (WB)	12	−76	24	3.7	29	<0.001
	Precentral gyrus L (WB)	−22	−14	62	3.67	26	<0.001
	Caudate body L (WB)	−18	20	8	3.53	7	<0.001
	Medial orbital frontal gyrus R (WB)	12	58	−12	3.43	5	<0.001
	Middle occipital gyrus L (WB)	−34	−66	18	3.32	7	<0.001
	Insula R (ROI)	44	2	0	3.01	10	0.001
	Caudate L (ROI)	−18	20	8	3.53	17	<0.001
	Putamen R (ROI)	36	−12	−8	3.07	14	<0.001
	Medial orbital frontal gyrus R (ROI)	12	58	−12	3.43	11	<0.001
Trace	Postcentral gyrus L (WB)	−42	−32	54	4.7	6	<0.001
	Rolandic operculum R (WB)	42	−22	26	4.37	86	<0.001
	Putamen L (WB)	−30	−14	2	4.11	8	<0.001
	Calcarine R (WB)	12	−92	12	4.09	19	<0.001
	Middle frontal gyrus R (WB)	40	6	40	3.55	7	<0.001
	Ventral ACC L (WB)	−12	10	30	3.55	8	<0.001
	Insula R (ROI)	36	−18	22	3.78	15	<0.001
	Dorsal ACC R (ROI)	4	22	34	2.93	11	0.002
	Putamen L (ROI)	−30	−14	2	4.11	14	<0.001
	Middle frontal gyrus R (ROI)	40	6	40	3.55	12	<0.001

α < 0.001 uncorrected for whole brain analysis (WB) and α < 0.005, uncorrected ROI analyses, with a minimum cluster size of k = 5 (WB) or k = 10 (ROI); L, left; R, right hemisphere. The cluster with the largest number of significant voxels within each region is reported. Coordinates x, y, and z of the peak voxels are given in Montreal Neurological Institute space.

#### Late extinction

***ROI analysis***. For the second phase of extinction, we observed significant activation in the ventral part of the ACC in the DCG only.

***Whole brain analysis***. In the DCG, the ventral ACC (R), the inferior frontal gyrus (R), and the supramarginal gyrus (R) were activated during late extinction. In the TCG however, we found significant activation of the precuneus (L and R).

For exact coordinates see Table 2.

**Table 2:** **Significant activations revealed by whole brain (WB) and regions of interest (ROI) analysis for contrast CS+ > CS− during late extinction**.

**Group**	**Brain structure**	***x***	***y***	***z***	***Z***	**Cluster size**	***P***
Delay	ventral ACC R (WB)	6	10	30	4.84	13	<0.001
	Triangular part of inferior frontal gyrus R (WB)	50	18	14	3.67	35	<0.001
	Supramarginal gyrus R (WB)	60	−34	28	3.66	25	<0.001
	Ventral ACC R (ROI)	6	10	30	4.84	17	<0.001
Trace	Precuneus R (WB)	14	−58	24	3.74	62	<0.001
	Precuneus L (WB)	−10	−62	30	3.37	10	<0.001
	ROI analysis: no significant voxel						

α < 0.001 uncorrected for whole brain analysis (WB) and α < 0.005, uncorrected ROI analyses, with a minimum cluster size of k = 5 (WB) or k = 10 (ROI); L, left; R, right hemisphere. The cluster with the largest number of significant voxels within each region is reported. Coordinates x, y, and z of the peak voxels are given in Montreal Neurological Institute space.

#### Early extinction > late extinction

***ROI analysis***. We also tested for areas, which showed stronger activation in the early extinction than in the late extinction. In the delay group, this was the case for the insula (L), whereas in the trace group the hippocampus (R) and the striatum (putamen L) showed greater activation in the first part of the extinction compared to the second part.

***Whole brain analysis***. Whole brain analysis revealed additional activation of the ACC (ventral anterior cingulate area), the precentral gyrus (L), and the transverse temporal gyrus (Heschl L) in the DCG. In the TCG, the ventral ACC (L) was activated as in the delay group, and additionally we found activation in the parahippocampal area.

For exact coordinates see Table 3.

**Table 3:** **Early extinction (CS+ > CS−) > late extinction (CS+ > CS−): significant activations revealed by whole brain (WB) and regions of interest (ROI) analysis**.

**Group**	**Brain structure**	***x***	***y***	***z***	***Z***	**Cluster size**	***P***
Delay	Precentral gyrus L (WB)	−22	−14	62	4.10	59	<0.001
	Ventral ACC L (WB)	−16	0	44	3.65	14	<0.001
	Heschl L (WB)	−32	−28	16	3.29	5	0.001
	Insula L (ROI)	−38	−20	14	3.05	25	0.001
Trace	Putamen L (WB)	−30	−14	2	3.74	13	<0.001
	Ventral ACC L (WB)	−10	14	30	3.61	10	<0.001
	Parahippocampus R (WB)	32	−34	−12	3.51	11	<0.001
	Hippocampus R (ROI)	30	−32	−8	3.87	11	<0.001
	Putamen L (ROI)	−30	−14	2	3.74	23	<0.001

α < 0.001 uncorrected for whole brain analysis (WB) and α < 0.005, uncorrected ROI analyses, with a minimum cluster size of k = 5 (WB) or k = 10 (ROI); L, left; R, right hemisphere. The cluster with the largest number of significant voxels within each region is reported. Coordinates x, y, and z of the peak voxels are given in Montreal Neurological Institute space.

## Discussion

To our knowledge, this is the first study investigating neural substrates of extinction of both delay and trace fear memory in humans. In both conditioning groups we found activation in the insular cortex and the striatum during early extinction. Interestingly, extinction of delay and trace memory differed in prefrontal activation. The vmPFC was activated during extinction in the DCG, while the dlPFC was activated during extinction in the TCG. These results point to different PFC activity involved in early extinction of delay vs. trace fear conditioning. In the late part of the extinction process, the delay group only showed significant activation of the ventral ACC. No other activation could be found in our predefined regions of interest during the second half of extinction. However, when comparing the early with the late part of extinction, we found greater activation in the insula (delay group), the hippocampus, and the striatum (trace group) during early extinction.

### Prefrontal cortex

The most prominent result of our study is the dissociation of prefrontal activation in delay vs. trace conditioning during early extinction. As has been shown in previous human fear conditioning studies, the vmPFC plays an important role in the extinction of fear memory (e.g., Phelps et al., 2004). In accordance with findings from the animal model it is assumed that, during extinction, an inhibitory memory trace is formed between the vmPFC and the amygdala (Sotres-Bayon et al., 2004, 2007), which allows for the modulation of the fear response. However, existing evidence from human studies for this model comes exclusively from delay fear conditioning. Significant activation of the vmPFC in the DCG of our study provides further evidence for this model. Milad et al. (2005) indicated that the vmPFC is not only involved in extinction learning, but also in the retention of extinction memory. They reported a significant correlation between the thickness of the medial orbitofrontal cortex and skin conductance response (SCR) in extinction recall assessed one day after extinction training. More precisely, a thicker medial orbitofrontal cortex was associated with a lower SCR in extinction recall, that is, with greater extinction memory.

However, in the TCG, we did not find significant activation of the vmPFC, but instead of the dlPFC. This finding points to different processes during extinction in delay and trace conditioning. VmPFC activation in delay conditioning reflects the inhibition of the fear response already during early extinction. According to a model of functional organization of the lateral PFC, the vmPFC is mainly involved in the mere maintenance of information, whereas the dorsal part is assumed to be involved in the manipulation of information, requiring more working memory capacities (D'Esposito et al., 1999; Postle et al., 1999). As mentioned in the introduction, lesion studies in nonhuman and human primates indicate that the dlPFC is important for adjusting behavior appropriately in delayed response tasks (e.g., D'Esposito et al., 2000), in which information has to be kept in working memory for a short period of time before making choices and decisions on the basis of this information. This interpretation is in line with activation of the dlPFC during the extinction of trace conditioning. In contrast to delay conditioning, trace conditioning and its extinction afford higher working memory contribution to bridge the trace interval and hold information in short term memory. Results of ratings indicate that extinction proceeded more slowly in the trace group compared to the delay group. In the DCG, we did no longer find differences in arousal and fear ratings of the CS+ compared to the CS− after extinction. However, the CS+ was still rated more arousing and more frightening than the CS− in the TCG. A slower extinction process in the trace group can be seen as an indication for a higher working memory contribution in the extinction of trace conditioning and therefore may account for different processes of extinction in delay and trace conditioning. There is also an interesting connection between our findings and evidence from trace eyeblink conditioning in rabbits (Weiss and Disterhoft, 2011). They assume an important role of the dlPFC in the acquisition of trace conditioning: Activation of dlPFC and hippocampus potentiates the effect of the CS at pontine nuclei on the way to the cerebellum and thus bridge the trace interval during acquisition. After consolidation of the CS+/US association, structures mediating the conditioned response reorganize. While the hippocampus becomes less important, the dlPFC becomes more important. Further research with regard to both acquisition and extinction is necessary for investigating to what extend these findings from the animal model can be transferred to classical conditioning in humans.

### Insula and ACC

In both conditioning groups we found activation of the insular cortex during early extinction. Additionally, the insula showed greater activation in the early compared to the late extinction in the delay group. Evidence for the involvement of the insula in classical fear conditioning comes, among others, from Phelps et al. (2001): In contrast to the instructed fear paradigm, in which the insula was activated already in early trials, the activation in the conditioning paradigm occurred not until the later trials of acquisition, when participants were consciously aware of the association between the CS+ and the US. These findings are consistent with evidence coming from pain research showing that the insula plays an important role in the anticipation of pain (e.g., Ploghaus et al., 1999; Wiech et al., 2010). Phelps et al. (2004) suggest that the anticipation of pain leads to a cortical representation of fear, which is transmitted to the amygdala via the insular cortex. During early extinction of fear memory, the CS+ is no longer followed by a painful stimulus. However, it is still associated with the US and thus leads to the anticipation of pain, resulting in the observed activation of the insular cortex. Interestingly, during extinction of both trace and delay fear conditioning, insular activation was limited to the right hemisphere. The left insula has been associated with semantic processing which is necessary in instructed fear conditioning. In contrast, the right insula has been associated with the response to a sensory aversive US like for example the mildly painful electric stimulus we applied (for further information see for example Craig, 2010).

In addition to the insula, the dorsal ACC was activated during early extinction in the TCG. Moreover, we found stronger activation of the ACC during early extinction compared to late extinction in both groups. The combined activation of the ACC and the insula has been discussed to represent a pathway for the integration of nociceptive input in memory processes (Coghill et al., 1994). According to this model, both structures are involved in the adjustment of behavior in response to a stimulus predicting pain (see also Büchel et al., 1998). The ACC has also been associated with sustained attention toward a stimulus that might be followed by pain (Yaguez et al., 2005). There is broad evidence that sustained attention is necessary for trace fear conditioning, but not for delay conditioning. In a trace conditioning paradigm, fear memory is only established when subjects are consciously aware of the CS–US contingency. However, in delay paradigm, conditioning can also occur when participants have not formed declarative memory and thus are unaware of the association between the CS and the US (e.g., Manns et al., 2001; Clark et al., 2002; Weike et al., 2007). Han et al. (2003) have shown that attention-distracting stimuli interfere with trace but not delay or contextual fear conditioning in mice. Moreover, they found a higher density of c-fos-positive cells in the ACC of mice that had undergone trace fear conditioning compared to delay conditioning. In the same study, lesions of the ACC selectively impaired trace conditioning. These results give additional evidence for the association of ACC activation and sustained attention during trace fear conditioning, and also offer an explanation why we found combined activation of the insula and the ACC only during the early extinction of trace but not delay fear memory.

### Striatum

During acquisition of fear memory, the expectation is formed that an initially neutral stimulus is followed by a negative event such as an electric stimulus. During extinction, this expectation is violated: The negative event does no longer occur. This discrepancy between the expected and the actual outcome is referred to as prediction error (e.g., Schultz et al., 1997). The striatum has been shown to be involved in the coding of prediction errors in both appetitive and also aversive classical and instrumental conditioning. This applies to primary reinforcers such as pain (Phelps et al., 2004; Seymour et al., 2005), but also to secondary ones such as monetary gains (e.g., Delgado, 2007). In our study, we found striatal activation in both the DCG and the TCG. These results provide further evidence for the important role of the striatum not only in the acquistion (Jensen et al., 2003; Delgado et al., 2008; Klucken et al., 2009; Tabbert et al., 2011), but also in the extinction of fear memory and therefore in the coding of prediction errors characterized by the omission of a negative outcome. Raczka et al. (2011) recently showed that a functional polymorphism of the dopamine transporter gene, which is mainly expressed in the striatum, influences extinction learning. The 9-repeat allele is associated with enhanced phasic dopamine release and with higher learning rates in the extinction of conditioned fear. In 9R carriers they also found stronger activation of the ventral striatum in response to prediction errors during extinction. In relation to these findings they assumed that extinction is an appetitive-like learning process mediated by the mesostriatal dopamine system, rather than a learning process driven by an aversive prediction error.

### Hippocampus

The hippocampus has been found to be involved in the representation of the temporal context in a conditioning process, which plays an important role in a trace paradigm including a temporal gap between CS+ and US (e.g., Phillips and LeDoux, 1992). In the TCG, we found greater hippocampal activation in the comparison of early vs. late extinction. Clark et al. (2002) stated that the hippocampus is crucial for explicit or declarative memory processes. According to them, trace conditioning requires declarative knowledge and therefore hippocampus activity because the temporal gap between the CS and the US makes it difficult to process the CS–US relationship in an automatic, reflexive way. Knight et al. (2004) found hippocampal activation during extinction as well. They also reported a rapid decrease of its activation during the early trials of extinction.

### Amydala

We did not find significant activation of the amygdala in either one of the two groups. One possible reason for this is the rapid habituation of amygdala activity during extinction learning, especially in a conditioning paradigm with 100% contingency of CS+ and US during acquisition (e.g., LaBar et al., 1998). Secondly, EPI is highly vulnerable to susceptibility artifacts, which occur near the interfaces of substance of different magnetic susceptibility and thus are likely in structures of the medial temporal lobe, like the amygdala (Bellgowan et al., 2006; Stocker et al., 2006).

### Conclusion

In sum, our results add further evidence for the involvement of the PFC, insula, ACC, striatum, and hippocampus in the extinction of conditioned fear memory. We could also confirm that the ACC and the hippocampus are mainly involved in trace conditioning processes. The ACC has been associated with sustained attention, which is necessary for trace but not delay conditioning. The hippocampus is assumed to be necessary for the processing of the temporal context necessary to bridge the trace interval. Most important, our results indicate that different parts of the PFC are activated during extinction of delay vs. trace fear conditioning, the vmPFC vs. the dlPFC, respectively. These results point to different underlying processes during extinction of these two types of conditioning. Due to limited power of our study and a relatively liberal level of significance, results have to be interpreted with care. More evidence is needed to elucidate the role of the PFC in the extinction of trace conditioning in more detail and to translate results from the animal model to human trace fear conditioning

### Conflict of interest statement

Prof. Paul Pauli and Prof. Andreas Mühlberger are Shareholders of a commercial company that develops virtual environment research systems for empirical studies in the field of psychology, psychiatry, and psychotherapy. Mathias Müller is shareholder and executive of the same company. The authors declare that the research was conducted in the absence of any commercial or financial relationships that could be construed as a potential conflict of interest.
